# Histone Deacetylase Inhibitor, Sodium Butyrate-Induced Metabolic Modulation in *Platycodon grandiflorus* Roots Enhances Anti-Melanogenic Properties

**DOI:** 10.3390/ijms241411804

**Published:** 2023-07-22

**Authors:** Min-A Ahn, Jinsu Lee, Tae Kyung Hyun

**Affiliations:** 1 Department of Industrial Plant Science and Technology, College of Agriculture, Life and Environment Sciences, Chungbuk National University, Cheongju 28644, Republic of Korea; koala0523@naver.com; 2 School of Biological Sciences, Seoul National University, Seoul 08826, Republic of Korea; jinsulee90@gmail.com

**Keywords:** anti-melanogenic property, histone acetylation, histone deacetylase inhibitor, transcriptome, *Platycodon grandiflorus*

## Abstract

While the status of histone acetylation is a critical regulator of chromatin’s structure with a significant impact on plant physiology, our understanding of epigenetic regulation in the biosynthesis of active compounds in plants is limited. In this study, *Platycodon grandiflorus* was treated with sodium butyrate (NaB), a histone deacetylase inhibitor, to investigate the influence of histone acetylation on secondary metabolism. Its treatment with NaB increased the acetylation of histone H3 at lysine 9, 14, and 27 and enhanced the anti-melanogenic properties of *P. grandiflorus* roots. Through transcriptome and differentially expressed gene analyses, we found that NaB influenced the expression of genes that were involved in both primary and secondary metabolic pathways. In addition, NaB treatment caused the accumulation of polyphenolic compounds, including dihydroquercetin, gallic acid, and 2,4-dihydroxybenzoic acid. The NaB-induced transcriptional activation of genes in the phenylpropanoid biosynthetic pathway influenced the anti-melanogenic properties of *P. grandiflorus* roots. Overall, these findings suggest the potential of an epigenomic approach to enhance the medicinal qualities of medicinal plants.

## 1. Introduction

Secondary metabolism in higher plants is a specialized process for producing metabolites that mediate the interaction between plants and their environmental conditions [1]. The mechanism underlying stress-induced secondary metabolism indicates that secondary metabolite accumulation is mainly mediated by the induction of key synthesis-related genes [2]. Moreover, transcriptional regulation by transcription factors can considerably influence secondary metabolism [3], which suggests that understanding upstream regulators is vital for the control and biosynthesis of secondary metabolites.

Epigenetic modifications regulate physiological and biochemical responses through the control of gene expressions without changing the DNA sequence [4]. Various plants, including maize, rice, tobacco, and Chinese cabbage, have exhibited dynamic changes in their DNA and histone modifications under environmental stress conditions, which influences metabolic dynamics [5,6,7,8,9,10]. Among these modifications, histone acetylation in different lysine residues of the histone plays a crucial role as an epigenetic regulatory mechanism for gene transcription, influencing numerous physiological and developmental processes in plants [4]. In addition, the levels of histone 3 and 4 acetylation in the promoter region of *Camellia sinensis* α-farnesene synthase increased in response to wounding stress [11]. In Arabidopsis histone deacetylase, 15-deficient mutants, including sucrose, induced histone H4 acetylation in the promoter and/or the exon regions of anthocyanin biosynthetic genes [12]. Similarly, the inhibition of histone deacetylase activities enhanced the production of methyl jasmonate (MeJA)-induced ginsenoside by increasing the expression levels of ginsenoside biosynthetic genes in ginseng adventitious roots [13].

The roots of *Platycodon grandiflorus*, as a monotypic species of the Campanulaceae family, contain considerable triterpenoid glycosides, flavonoids, phenolic acids, and sterols with broad pharmacological activities, including anti-inflammatory and anti-melanoma properties [14]. MeJA has been widely used to improve the production of secondary metabolites in *P. grandiflorus* roots and its hairy roots [15,16]. Thus, MeJA is known as an epigenetic modulator [13,15]. For example, MeJA leads to the enrichment of histone acetylation on specific genes involved in α-linolenic acid metabolism and phenylpropanoid biosynthesis [17]. Taken together, this suggests that manipulating the acetylation status of histones is a viable approach for enhancing secondary metabolite production.

In this study, to investigate the effect of histone acetylation on the quality-related metabolism in *P. grandiflorus* roots, we analyzed the anti-melanoma property of *P. grandiflorus* roots when treated with sodium butyrate (NaB) as a histone deacetylase inhibitor. The analysis of differentially expressed genes (DEGs) and metabolites in NaB-treated roots revealed the involvement of NaB in phenylpropanoid metabolism. Overall, our results provide key insights into the contribution of histone acetylation on the secondary metabolism of *P. grandiflorus* roots.

## 2. Results and Discussion

### 2.1. Effect of NaB on Histone 3 Acetylation (H3ac) in P. grandiflorus Roots

**Figure 1:**
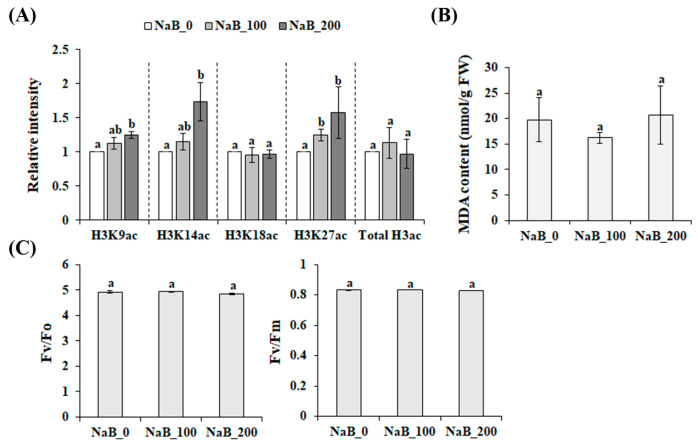
Physiological response to NaB treatment in *P. grandiflorus*. (**A**) The acetylation status of histone H3 in NaB-treated *P. grandiflorus* roots. Changes in the levels of MDA (**B**) and photosynthesis (**C**) after NaB treatment was determined. The potential activity of PSII (Fv/Fo) and the maximum quantum efficiency of PS II photochemistry (Fv/Fm) were determined using a pulsed modular fluorometer. Means (±SE) with different letters (*p* < 0.05, Duncan’s multiple range test) are significantly different. NaB_0, non-treated samples; NaB_100, samples treated with 100 µM NaB; NaB_200, samples treated with 200 µM NaB.

The acetylation of lysine residues on the N-terminal tail of H3 by histone acetyltransferases increased the accessibility of transcription factors by opening or closing the chromatin structure, whereas histone deacetylases removed the acetyl groups from hyperacetylated histones [13,18]. Thus, the treatment with histone deacetylase inhibitors, including NaB, led to histone hyperacetylation in higher plants [13,19,20]. Similarly, the H3ac on lysine 9 (H3K9ac), lysine 14 (H3K14ac), and lysine 27 (H3K27ac) increased significantly after five days of exposure to NaB; however, NaB treatment did not affect the level of H3ac on lysine 18 (H3K18ac) and the total H3ac in *P. grandiflorus* roots (Figure 1A). In higher plants, H3K9ac, H3K14ac, and H3K27ac were associated with transcriptional activation [21,22,23,24], indicating that our treatment was sufficient for inducing the transcriptional and chromatin changes in *P. grandiflorus* roots. In human colorectal cells, NaB induced endoplasmic reticulum (ER) stress, which caused the accumulation of misfolded or unfolded proteins in the ER [25]. However, ER stress in plants can lead to oxidative stress, which causes photosynthesis impairment, autophagy, and cell death [26,27]. This suggests that NaB treatment probably caused the induction of ER stress, which negatively affected plant growth and development. To test this hypothesis, we determined the effect of NaB treatment on photosynthesis and MDA content, which is known as a lipid peroxidation indicator [28]. The NaB treatment did not induce MDA accumulation in P. grandiflorus roots (Figure 1B). Chlorophyll fluorescence parameters, including the potential activity of PSII (Fv/Fo) and the maximum quantum efficiency of PSII photochemistry (Fv/Fm), were unaffected by NaB treatment (Figure 1C). This indicates that NaB treatment did not induce ER stress, which caused lipid peroxidation and photosynthesis impairment.

### 2.2. Effect of NaB on the Anti-Melanogenic Properties of P. grandiflorus Roots

**Figure 2:**
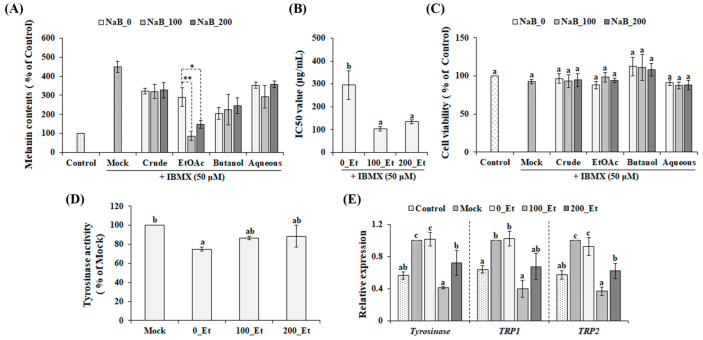
The effect of NaB treatment on anti-melanogenic properties of *P. grandiflorus* roots. The inhibitory effects (**A**) and cytotoxic effects (**C**) of crude extracts and their solvent fractions on IBMX-induced melanin production in B16F10 melanoma cells were analyzed. (**B**) IC50 values of the IBMX-induced melanin production of EtOAc fractions. (**D**) Inhibitory effect of EtOAc fractions on tyrosinase activity. (**E**) The effect of EtOAc fractions on the levels of melanogenesis-related genes in IBMX-stimulated B16F10 cells. Each gene transcription level in each sample was expressed relative to that of the mock control (Mock). Means (±SE, three independent experiments) with asterisks (* *p* < 0.05 and ** *p* < 0.01, *t*-test) or different letters (*p* < 0.05, Duncan’s multiple range test) are significantly different. 0_Et, EtOAc fractions of the extract obtained from non-treated samples; 100_Et, EtOAc fractions of the extract obtained from 100 µM NaB-treated samples; 200_Et, EtOAc fractions of the extract obtained from 200 µM NaB-treated samples; TRP1, tyrosinase-related protein 1; TRP2, tyrosinase-related protein 2.

To investigate the effect of H3 hyperacetylation on the medicinal qualities of *P. grandiflorus* roots, NaB-induced variation in the anti-melanogenic properties was determined using B16F10 melanoma cells. The anti-melanogenic properties of 70% EtOH extracts from NaB-treated (100 μM, NaB_100; 200 μM, NaB_200) and non-treated (NaB_0) samples were found to be similar in 3-isobutyl-1-methylxanthine (IBMX)-stimulated B16F10 cells (Figure 2A). However, after solvent partitioning, the EtOAc fractions contained significantly different anti-melanogenic properties without cytotoxic activities against B16F10 melanoma cells (Figure 2A,C). In addition, EtOAc fractions of NaB_100 and NaB_200 exhibited the highest anti-melanogenic properties compared with other fractions (Figure 2A). The anti-melanogenic properties of EtOAc fractions obtained from NaB_100 (100_Et; IC50 = 104.0 ± 9.2 µg/mL) or NaB_200 (200_Et; IC50 = 134.3 ± 8.2 µg/mL) were significantly greater than that of the EtOAc fraction of NaB_0 (0_Et; IC50 = 294.7 ± 63.5 µg/mL) (Figure 2B), which indicated that NaB improved the anti-melanogenic properties of *P. grandiflorus* roots. Furthermore, the anti-melanogenic properties of 100_Et and 200_Et were mediated by inhibiting the expression of melanogenic enzymes, including tyrosinase and tyrosinase-related protein 1 and 2 (Figure 2E), rather than the inhibition of tyrosinase activity (Figure 2D). Overall, these findings suggest that NaB-induced H3 hyperacetylation improved the production of phytochemicals, which could suppress the expression of melanogenic enzymes in IBMX-stimulated B16F10 cells.

### 2.3. Effect of NaB Treatment on Gene Expression in P. grandiflorus Roots

**Figure 3:**
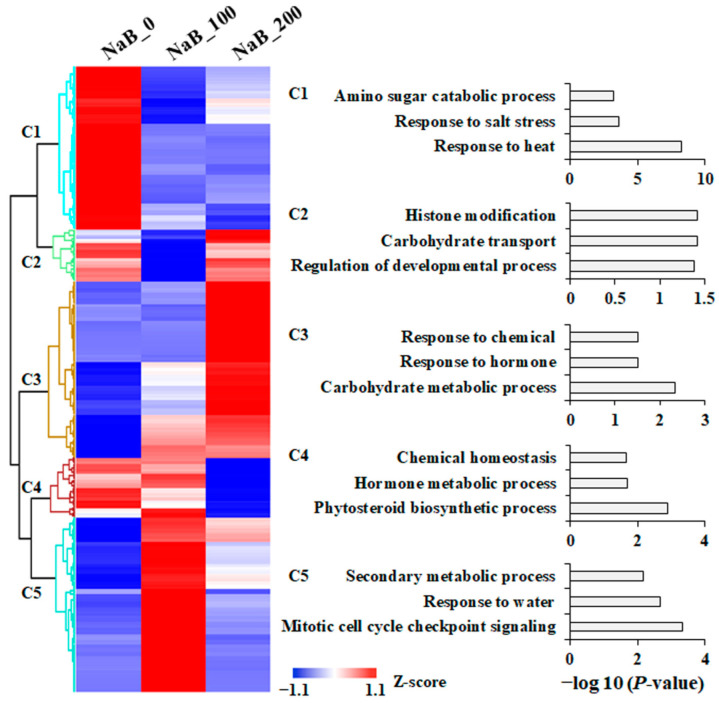
NaB-induced differentially expressed genes (DEGs) in *P. grandiflorus* roots. Hierarchical analysis was conducted according to the gene expression patterns of DEGs, and gene ontology enrichment analysis for the biological processes was performed for DEGs in each cluster.

H3acs, especially H3K9ac, H3K14ac, and H3K27ac, were associated with transcriptional activation in higher plants [21,22,23,24], which indicated that the NaB-improved anti-melanogenic property was mediated by transcriptional changes. To investigate the effects of NaB treatment on the transcription in *P. grandiflorus* roots, cDNA libraries from the NaB_0, NaB_100, and NaB_200 were sequenced. After the removal of low-quality reads and adaptor sequences, 50.2 to 56.4 million clear reads (7.29 to 8.29 Gb) obtained from each sample were used for the analysis of DEG (Table 1). Two pair-wise comparisons (NaB_0 vs. NaB_100 and NaB_0 vs. NaB_200) were conducted to identify NaB-induced DEGs. According to the comparison between NaB_0 and NaB_100, a total of 1213 genes (423 up-regulated and 790 down-regulated) were identified as DEGs. Moreover, 415 genes were identified as up-regulated, and 318 genes were down-regulated in NaB_200 (Figure S1). Using hierarchical clustering expression patterns for all 1248 DEGs, we identified five clusters (C1 to C5) (Figure 3 and Figure S2) and analyzed the gene ontology (GO) term. C3 (top GO terms in the biological process: “response to chemical,” “response to hormone,” and “carbohydrate metabolic process”) was up-regulated via NaB treatment, whereas C1 (“amino sugar catabolic process,” “response to salt stress,” and “response to heat”) was down-regulated via NaB treatment (Figure 3 and Figure S2). Similar to these findings, the genes belonging to a “response to stimulus” and “primary metabolic process” were up-regulated in SAHA (histone deacetylase inhibitor)-treated cassava [29]. In addition, NaB treatment affected amino acid and carbohydrate metabolisms during the germination of *Medicago truncatula* [30]. In *Brassica napus* microspore cultures, auxin-related genes were up-regulated via the treatment of trichostatin A (TSA; histone deacetylase inhibitor) [31]. Furthermore, the C5 group represented genes that were highly upregulated in NaB_100 and enriched in the “secondary metabolic process,” which included the phenylpropanoid and lignin metabolic processes (Figure 3). In *Malus crabapple*, TSA treatment induced anthocyanin accumulation via the induction of anthocyanin metabolic genes [32]. These findings indicate that histone deacetylase-dependent mechanisms contribute to the regulation of primary and secondary metabolic pathways in various plants, including *P. grandiflorus*.

### 2.4. Effect of NaB on the Phenylpropanoid Biosynthetic Pathway in P. grandiflorus Roots

**Figure 4:**
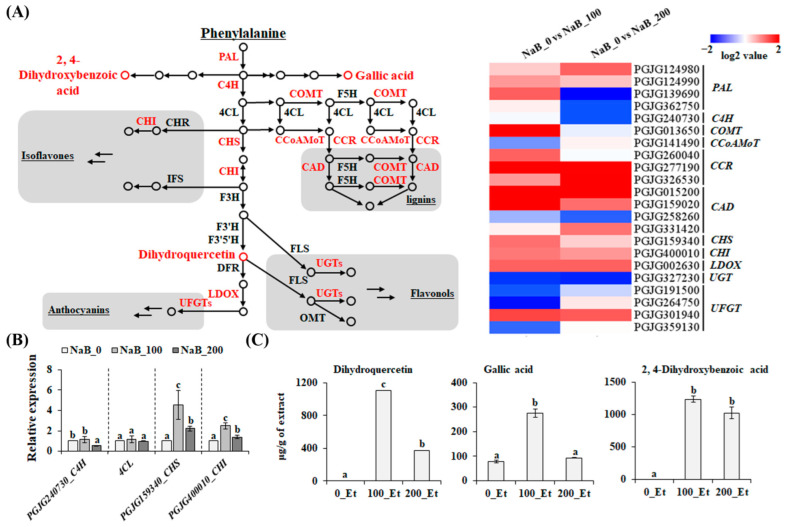
The effect of NaB treatment on the phenylpropanoid pathway. (**A**) NaB-induced DEGs involved in the biosynthesis of phenylpropanoids. (**B**) qRT-PCR validation using selected genes. (**C**) The levels of dihydroquercetin, gallic acid, and 2, 4-dihydroxybenzoic acid were assessed using HPLC. Means (±SE, three independent experiments) with different letters (*p* < 0.05, Duncan’s multiple range test) are significantly different. 0_Et, EtOAc fractions of the extract obtained from non-treated samples; 100_Et, EtOAc fractions of the extract obtained from 100 µM NaB-treated samples.

While triterpenoid saponins, such as platycodin D, have been recognized as the major active compound in *P. grandiflorus* roots [14], further phytochemical investigations have revealed that polyphenolic compounds are also essential active components in *P. grandiflorus* roots [14,33]. In addition, the EtOAc fraction has been proposed as the optimal method for concentrating active polyphenols in plant extracts [34]. Hence, we hypothesized that NaB treatment enhanced the anti-melanogenic properties of *P. grandiflorus* roots by promoting phenylpropanoid biosynthesis. As shown in Figure 4A, the NaB treatment upregulated a number of genes that were involved in phenylpropanoid biosynthesis, although the expression levels varied depending on the NaB concentration used (Figure 4A). Selected genes were subjected to qRT-PCR analysis which validated RNA-seq data and suggested that the results of our RNA-seq experiments were reliable (Figure 4B). To determine whether the up-regulation of these genes correlated with an accumulation of polyphenolic compounds, we quantified the abundance of dihydroquercetin, gallic acid, and 2,4-dihydroxybenzoic acid. In NaB_100, the levels of these compounds were significantly higher than those in NaB_0 (Figure 4C). Similarly to the expression patterns (Figure 4A), the accumulation of these compounds also varied depending on the NaB concentration used (Figure 4C). While a slight difference in anti-melanogenic properties existed between NaB_100 and NaB_200 (Figure 1A), the variation in the accumulation of these compounds could explain the difference in their activities. Similar to our findings, previous studies have also reported the dose-independent effects of NaB. For instance, in grapevine, embryogenic responses were enhanced with a 0.5 mM NaB treatment, while 2 mM NaB inhibited these responses [35]. Likewise, in wheat, regeneration efficiency was improved with a 200 µM NaB treatment, demonstrating superior effectiveness compared to 1000 µM of NaB [36]. These studies provide further evidence to support the existence of the dose-independent effects of NaB. Overall, these findings indicate that NaB improved the anti-melanogenic properties of *P. grandiflorus* roots by promoting the production of active compounds, including polyphenols.

## 3. Materials and Methods

### 3.1. NaB Treatment and Preparation of Extracts

One-year-old roots of *P. grandiflorus* (cultivar Jangbaek-doraji) were cultured under controlled conditions [24 °C, long photoperiod (16 h light/8 h dark), and 50% relative humidity], and four-week-old plants were watered with a NaB solution of 100 to 200 µM. After five days of treatment, the roots were harvested, frozen in liquid nitrogen, and kept at −80 °C for further analysis. Extracts were prepared using 70% EtOH. The extracts were then used to prepare fractions of ethyl acetate, n-butanol, and aqueous fractions, as described by Kim and Hyun [33]. The 70% EtOH extract and its fractions were re-dissolved in dimethylsulfoxide for further analysis.

### 3.2. Determination of Histone H3 Acetylation Pattern

The histone-enriched extracts were prepared, as described by Eom and Hyun [8]. To detect the pattern of histone H3 acetylation, 10 μg of nuclear proteins were separated into 15% SDS-polyacrylamide gel and immunoblotted with specific antibodies, as described by Eom and Hyun [8]. A chemiluminescence system was used to visualize the signal, and ImageJ was utilized to analyze the relative intensity (the ratio of acetylated histone H3 to the total histone H3).

### 3.3. Analysis of Photosynthesis and Malondialdehyde Contents

The effects of NaB treatment on photosynthesis were determined through the analysis of chlorophyll fluorescence using a pulsed modular fluorometer (FluorPen FP110, Photon Systems Instruments, Drásov, Czech Republic).

The malondialdehyde (MDA) content was determined using a colorimetric assay and a thiobarbituric acid reaction and was expressed in nmol/mg of the fresh weight (FW). The MDA content was determined according to the absorbance coefficient of extinction (155 mM^−1^ cm^−1^) [37].

### 3.4. Analysis of Anti-Melanogenic Properties and Cytotoxicity

To determine the anti-melanogenic property, IBMX-stimulated B16F10 melanoma cells were treated with each sample. After 48 h of incubation in a CO_2_ incubator, the melanin content and cell viability were analyzed [38]. Tyrosinase inhibitory activities were analyzed using a tyrosinase inhibition screening kit (BioVision, Milpitas, CA, USA) according to the provided protocol.

### 3.5. Determination of Expression Levels of Melanogenic Enzymes

The total RNA was isolated from B16F10 cells using the TRIzol reagent, and the expression levels of each gene were determined using real-time PCR based on SYBR Green. The primers used are listed in Table S1.

### 3.6. HPLC Analysis

The content of the polyphenolic compounds in the ethyl acetate fractions was determined via HPLC coupled with a diode array detector [33]. The mobile phases consisted of 0.1% formic acid in distilled water (mobile phase A) and acetonitrile containing 0.1% formic acid (mobile phase B). The gradient was 0–0.01 min, 90% A; 0.01–28 min, 60% A; 28–39 min, 40% A; 39–50 min, 10% A; 50–55 min, 10% A; 55–56 min, 90% A; and 56–65 min, 90% A. The run time was 65 min using a flow rate of 0.7 mL/min. The concentration of the target compounds in these samples was calculated through the comparison of the peak areas of the samples with the calibration curves of known standards.

### 3.7. Transcriptome Analysis

To generate cDNA libraries, 200 ng of the total RNA from three independent replicates were combined, and paired-end sequencing was performed using the Illumina HiSeq™ 2500 sequencing platform. Sequencing results were deposited at the National Agricultural Biotechnology Information Center (http://nabic.rda.go.kr, accessed on 15 January 2023; Table 1). Clean reads were obtained following the described protocol [37] and were aligned to the *P. grandiflorus* genome sequence data [15]. The transcript levels of each gene were expressed as fragments per kilobase of the transcript sequence per million base pairs (FPKM), and DEGs were determined [37]. We used Blast2GO (https://www.blast2go.com/, accessed on 15 January 2023) to perform the functional classification of the identified DEGs.

## 4. Conclusions

In this study, we explored the involvement of histone acetylation in secondary metabolism. The results suggest that the variation in metabolic processes induced by NaB-mediated H3ac influenced the anti-melanogenic properties of *P. grandiflorus* roots. However, further investigations are required to determine the levels of H3acs, particularly H3K9ac, H3K14ac, and H3K27ac, in the phenylpropanoid biosynthetic genes. Our findings provide a valuable foundation to improve the biosynthesis of active compounds in medicinal plants through the modulation of epigenetic events.

## Figures and Tables

**Table 1:** Summary of RNA sequencing data obtained from non- and NaB-treated samples.

Sample ID	NaB Treatment	Clean Reads	Clean Bases (Gb)	Mapped Reads (%)	Accession Number (NABIC)
NaB_0	Non-treated	56,411,520	8.29	94.42	NN-8312
NaB_100	100 µM	50,028,868	7.29	94.12	NN-8313
NaB_200	200 µM	55,955,892	8.24	95.26	NN-8314

## Data Availability

The data presented in this study are available on request from the corresponding author. The data are not publicly available due to privacy reasons.

## Supplementary Materials

The following supporting information can be downloaded at: https://www.mdpi.com/article/10.3390/ijms241411804/s1.
