# Supplementary material for: Histone Deacetylase Inhibitor, Sodium Butyrate-Induced Metabolic Modulation in Platycodon grandiflorus Roots Enhances Anti-Melanogenic Properties
Source: Int J Mol Sci. 2023 Jul 22;24(14):11804. doi: 10.3390/ijms241411804 (PMC10380954; doi:10.3390/ijms241411804)
Supplement: Supplementary file 1 [file ijms-24-11804-s001.zip › ijms-2504191-supplementary.pdf]

Supplementary Information

Table S1: Primer list for qRT-PCR analysis

**Gene**	**Sequence (5'-3')**
Tyrosinase	F-ATAGGTGCATTGGCTTCTGG<br>R-CCAACGATCCCATTTTTCTT
TRP1	F-GAGTGACATCCTGTGGCTCA<br>R-CGATACCCTGGGAACACTTT
TRP2	F-GCATCTGTGGAAGGGTTGTT<br>R-ACTCCTTCCTGAATGGGACC
β-actin	F-CCCACTCCTAAGAGGAGGATG<br>R-AGGGAGACCAAAGCCTTCAT

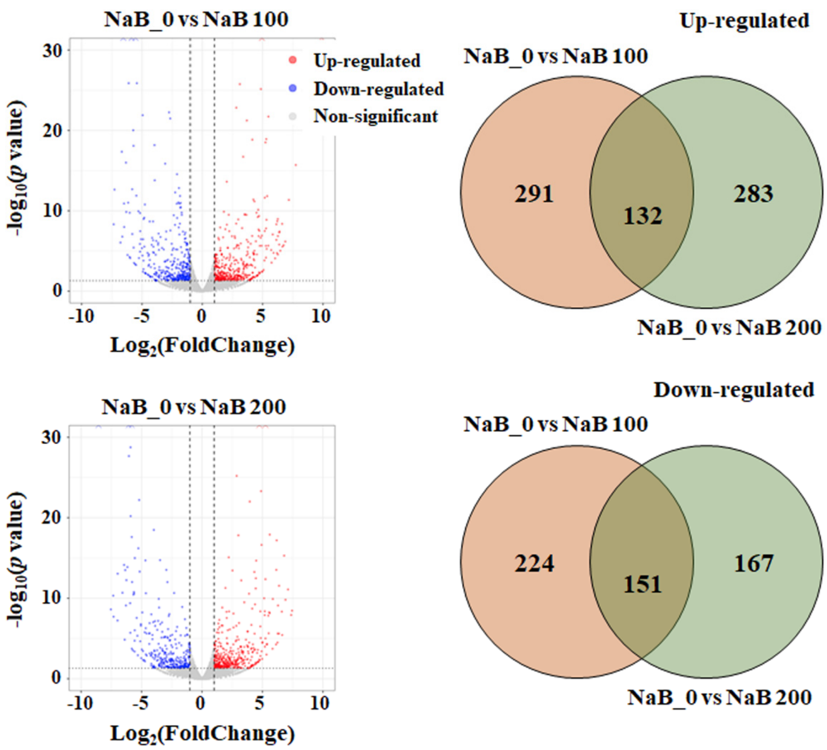

Figure S1. Volcano plot and venn diagram analyses of the DEGs (NaB_0 vs NaB 100 and NaB_0 vs NaB 200)

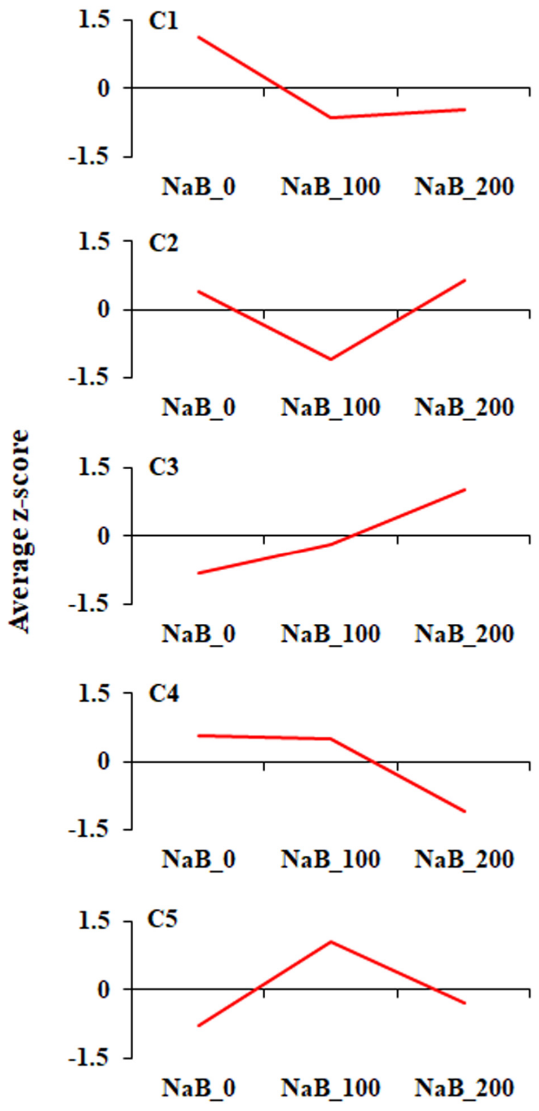

Figure S2. The lines reflect the expression patterns (average z-score) of DEGs in each cluster.
